# Monitoring response to gefitinib in nude mouse tumor xenografts by ^18^ F-FDG microPET-CT: correlation between ^18^ F-FDG uptake and pathological response

**DOI:** 10.1186/s12957-015-0505-x

**Published:** 2015-03-15

**Authors:** Li-Na Zhou, Ning Wu, Ying Liang, Kai Gao, Xiao-Ying Li, Lian-Feng Zhang

**Affiliations:** Department of Diagnostic Radiology, Cancer Hospital, Chinese Academy of Medical Sciences, No.17, Pan Jia Yuan Nan-li, Beijing, 100021 China; PET-CT Center, Cancer Hospital, Chinese Academy of Medical Sciences, No.17, Pan Jia Yuan Nan-li, Beijing, 100021 China; Key Laboratory of Human Disease Comparative Medicine, Ministry of Heath, Institute of Laboratory Animal Science, Peking Union Medical College, Chinese Academy of Medical Science, No.5, Pan Jia Yuan Nan-li, Beijing, 100021 China

**Keywords:** Xenograft, Fluorodeoxyglucose, Positron emission tomography, Tomography, Targeted therapy

## Abstract

**Background:**

The purpose of this study is to investigate whether ^18^ F-fluorodeoxyglucose (FDG) micro-positron emission tomography-computed tomography (microPET-CT) can be used to monitor a metabolic response to gefitinib in nude mouse tumor xenografts.

**Methods:**

Sixteen nude mice were implanted with human A431 epidermoid carcinoma cells and ten with human A549 lung adenocarcinoma cells, and the tumors were allowed to grow to an approximate size of 150 mm^3^. Ten and five of these mice, respectively, received intragastric gefitinib (100 mg/kg) once daily for 14 days, whereas six and five, respectively, received sterile water. Tumor metabolic activity was assessed by ^18^ F-FDG microPET imaging before treatment (day 0) and on days 2, 7, and 14. Tumor uptake of ^18^ F-FDG was determined from a region-of-interest drawn around the tumor, and the maximum percentage injected dose per gram (%ID/g_max_) was calculated. Tumor volume measured on day 14 by microCT was used to categorize tumors as sensitive, stable, or resistant to gefitinib, and pathologic changes in these tumors were analyzed.

**Results:**

On day 2, the average changes in ^18^ F-FDG uptake by A431 tumors sensitive, stable, and resistant to gefitinib were −30.92% ± 6.66%, −5.68% ± 6.95%, and 7.72% ± 3.85%, respectively (*P* < 0.05 each), with no significant differences in the sizes of tumors sensitive and stable to gefitinib (*P* = 0.169). On day 7, sensitive tumors were significantly smaller than stable tumors (*P* = 0.034). On day 14, areas of necrosis were observed in gefitinib-sensitive tumors, with tumor necrosis ratios differing significantly among the sensitive, stable, and control groups (*P* < 0.05 each). In mice implanted with A549 cells, however, tumor ^18^ F-FDG uptake, volume, and percent necrosis did not differ significantly between gefitinib-treated and untreated mice on days 0, 2, 7, and 14 (*P* > 0.05 each).

**Conclusions:**

F-FDG uptake is a sensitive method of detecting metabolic changes in tumors associated with therapy *in vivo*.

## Background

Epithelial growth factor receptor tyrosine kinase inhibitors (EGFR-TKIs) have significant antitumor activities, with clinical benefits observed in patients with various tumor types [1-4]. Unfortunately, however, responses have been seen only in subgroups of patients [3,5], making it critical to select only those patients who could benefit from treatment with EGFR-TKIs.

Specific mutations in the K-RAS and EGFR genes (exons 18, 19, and 21) in tumors have been shown to be biomarkers of response to EGFR-TKI [6,7]. Although these genetic markers may be used to predict treatment outcome, these tests are invasive, unpleasant, and inconvenient for patients. During the course of treatment, it is difficult to repeatedly acquire appropriate tumor samples for monitoring responses to EGFR-TKI [8]. There is a clear clinical need for noninvasive, safe, real-time, quantitative, and readily available approaches to monitor therapeutic responses to treatment, thus improving patient management.

Positron emission tomography (PET) imaging, which shows the early effects of treatment on tumor metabolism, has been widely used in the clinical management of patients with cancer. ^18^ F-fluorodeoxyglucose (FDG) PET-computed tomography (PET-CT) has been used to measure metabolic responses of tumor to treatment, including chemotherapy, radiotherapy, and targeted therapy [9,10]. Indeed, clinical trials have shown that ^18^ F-FDG PET-CT imaging may be a reliable surrogate marker of early therapeutic responses and clinical benefits [11].

Treatment of non-small cell lung cancer (NSCLC) cell lines sensitive to the EGFR-TKI gefitinib with this agent resulted in a decline in ^18^ F-FDG uptake within 2 h, before the inhibition of cellular proliferation and the induction of apoptosis [12]. In addition, ^18^ F-FDG uptake by gefitinib-sensitive xenografts in mice began to decline as soon as 48 h after initial treatment with gefitinib. A recent evaluation in 20 patients with lung adenocarcinoma treated with gefitinib suggested that changes in tumor ^18^ F-FDG uptake may predict response and outcome [13]. Patients who later exhibited longer progression-free survival (PFS) showed a 20% standardized uptake value (SUV) decrease in ^18^ F-FDG uptake after 2 days of gefitinib therapy. A more recent study, assessing ^18^ F-FDG PET-CT in 23 patients with NSCLC who received neoadjuvant EGFR-TKI, found that metabolic response after 7 days could predict pathological response [14]. These results indicate a need for additional prospective studies to confirm whether change in tumor ^18^ F-FDG uptake after 2 days of treatment with an EGFR-TKI is an early sensitive marker of its effectiveness.

To investigate whether ^18^ F-FDG PET-CT can predict pathological responses after 2 days of gefitinib treatment and to determine the optimal cutoff value of ^18^ F-FDG uptake for detecting sensitivity to treatment, we assessed the relationship between gefitinib treatment and ^18^ F-FDG uptake, as well as between changes in ^18^ F-FDG uptake and tumor pathology in human tumor-bearing mice.

## Methods

### Animals

All animal experiments were approved by the PUMA Institutional Animal Research Committee. This study used 26 female Balb/c nude mice 6 to 8 weeks old and weighing 20 to 24 g.

### Xenograft models

The human A431 epidermoid carcinoma and A549 lung adenocarcinoma cell lines were generously provided by the Institute of Laboratory Animal Science, Chinese Academy of Medical Sciences & Comparative Medical Center, Peking Union Medical College. A431 cells, in which the wild-type EGFR gene is amplified and large amounts of the protein expressed, are sensitive to EGFR-TKIs [12]. In contrast, A549 cells, which express wild-type EGFR, have a Ras mutation responsible for resistance to EGFR-TKIs.

All cells were grown in DMEM containing 10% fetal calf serum, 100 IU/mL penicillin, and 100 μg/mL streptomycin at 37°C in an atmosphere containing 5% CO_2_. Each mouse was inoculated subcutaneously in the right flank with 2 × 10^7^ A431 or A549 cells.

### Experimental design

Sixteen mice were implanted with A431 cells and ten with A549 cells. After the tumors had grown to an approximate size of 150 mm^3^, each group was randomly divided into two subgroups. Ten A431-implanted and five A549-implanted mice were intragastrically administered 100 mg/kg/day gefitinib, whereas six and five mice, respectively, were administered sterile water, as control. ^18^ F-FDG microPET-CT was performed the day before gefitinib administration (day 0) and on days 2, 7, and 14. The mice were subsequently sacrificed, and the tumor tissues rapidly resected. Tumors that had decreased in size ≥20% on day 14 were regarded as sensitive group; those that remained unchanged in size or had increased slowly, to a size smaller than that in the control group, were regarded as stable; and those that had increased to a size not significantly different from that of the control group were regarded as resistant.

### ^18^ F-FDG microPET-CT imaging

^18^ F-FDG was kindly provided by the PET-CT Center, Cancer Hospital, Chinese Academy of Medical Sciences, Peking Union Medical College. The Siemens Inveon combined microPET-CT scanner (Siemens Preclinical Solution USA, Inc., Knoxville, TN, USA) was provided by the Institute of Laboratory Animal Science, Chinese Academy of Medical Sciences & Comparative Medical Center, Peking Union Medical College. MicroCT scans were performed with an X-ray tube voltage of 80 kV, a current of 500 μA, an exposure time of 130 ms, and 120 rotation steps. The mice were anesthetized with isoflurane (2% in 100% oxygen) and, 10 min later, were injected intravenously with 290 ~ 320 μCi of ^18^ F-FDG in 200 μL saline. Imaging was started 60 min later. During scanning, each mouse was placed prone on the examination bed, and isoflurane (2% in 100% oxygen), with anesthesia maintained and lasting for 20 min. All microPET data were reconstructed with the filtered back-projection reconstruction algorithm.

### Quantitative image analysis

Images were analyzed with the Inveon Research Workplace (Siemens, Erlangen, Germany). Regions of interest (ROIs) were drawn manually following qualitative assessment of the entire tumor. The maximal percentage of injected dose per gram of tissue (%ID/g_max_) was determined using ROIs drawn around areas of increased tracer accumulation on serial microPET images [15]. Tumor volume was determined by summation of voxels within the tomographic planes on serial CT images. The tumor volume response to therapy was expressed as ΔVolume_day *n*_ = [(Volume_day *n*_ − Volume_day 0_)/Volume_day 0_] × 100%, and the %ID/g_max_ response as Δ%ID/g_max day *n*_ = [(%ID/g_max day *n*_ − %ID/g_max day 0_)/%ID/g_max day 0_] × 100%, as described [15]. All images were traced by an investigator blinded to treatment assignment.

### Histopathologic examination

Following tumor resection, each was cut open across its maximum dimension, fixed in 10% formalin, embedded in paraffin, sectioned at 4 mm thick with a microtome (RM2255, Leica Biosystems, Nussloch, Germany), and stained with hematoxylin and eosin. Pictures taken at high magnification (×200) were analyzed using Image-Pro Plus 6.0 professional image analysis software [16]. On each histopathologic slide, five high-power fields were randomly chosen for counting (one each in the upper left, upper right, lower left, lower right, and middle). Necrotic areas were measured, and the necrotic fraction of each field was expressed as a percentage.

### Statistical analysis

Results were presented as mean ± SD. Tumor responses in each group were evaluated by ANOVA. Statistical analyses were performed to correlate the percent necrosis per specimen with the change in %ID/g_max day 14_. The areas under the receiver operating characteristic (ROC) curves (area under the curve, AUC) of %ID/g_max_ or Δ%ID/g_max_ on days 2, 7, and 14 were calculated by using a nonparametric method to assess whether an AUC of 0.5 for %ID/g_max_ or Δ%ID/g_max_ could discriminate between tumors sensitive and stable to gefitinib and to determine the possible AUC cutoff points for %ID/g_max_ and Δ%ID/g_max_. *P* values less than 0.05 were considered statistically significant. Data from A431 and A549 tumors were analyzed independently.

## Results

### Changes in tumor volume on microCT

**Figure 1:**
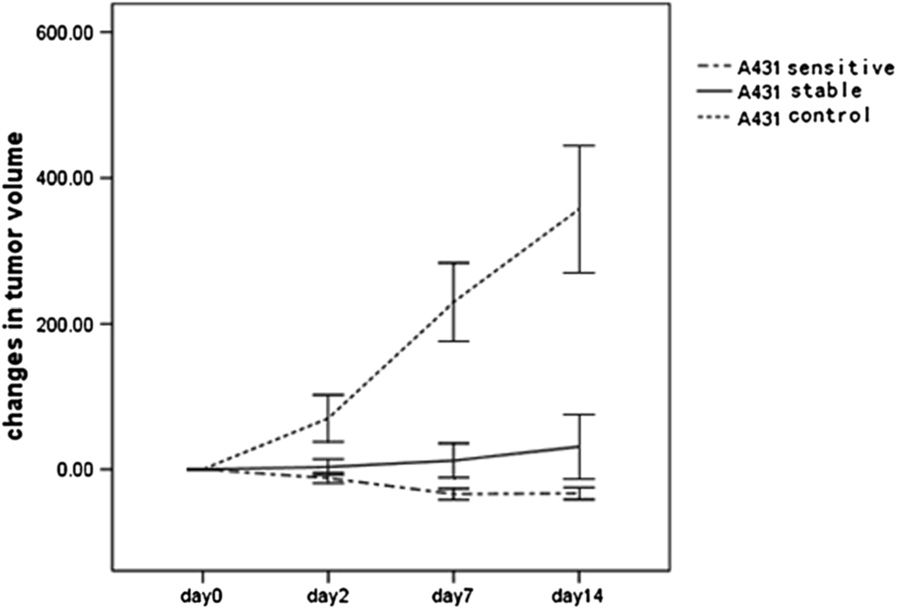
**Changes in A431 tumor volume during treatment with gefitinib.** No significant differences were seen among the groups on days 0 and 2 (*P* > 0.05). Significant differences among the groups were observed on days 7 and 14 (*P* < 0.05).

Before gefitinib treatment, there were no significant differences in the volumes of A431 (*n* = 16) and A549 tumors (*n* = 10) [(201.54 ± 40.38 mm^3^) *vs.* (213.40 ± 69.23 mm^3^), *P* = 0.092]. Measurement of A431 tumor volume after 14 days of gefitinib treatment showed that six of these tumors were sensitive, four were stable, and none was resistant to gefitinib treatment. Before treatment, the mean volume of tumors found to be sensitive (215.78 ± 33.57 mm^3^) and stable (193.45 ± 42.81 mm^3^) to gefitinib was similar to that in untreated mice (192.70 ± 42.71 mm^3^). On day 2, the changes in mean tumor volume in these three groups were −12.13% ± 6.79%, 3.22% ± 6.79%, and 70.28% ± 30.81%, respectively, with no significant difference between the sensitive and stable groups (*P* = 0.169). On day 7, the changes from baseline in mean tumor volumes were −34.01% ± 7.17%, 12.02% ± 14.92%, and 229.65% ± 50.85%, respectively, with gefitinib-sensitive tumors being significantly smaller than gefitinib-stable (*P* = 0.034) and untreated (*P* = 0.000) tumors. On day 14, mean tumor volumes in these three groups were 143.65 ± 21.73, 264.90 ± 73.35, and 852.48 ± 99.42 mm^3^, respectively, corresponding to mean changes from baseline of −32.97% ± 7.91% (*P* = 0.047), 35.91% ± 18.51% (*P* = 0.000), and 357.21% ± 83.09% (*P* = 0.000), respectively (Figure 1).

Examination of tumor volume in the mice implanted with A549 cells and treated for 14 days showed that none was sensitive or stable to gefitinib, whereas five were resistant. Before treatment, the mean volumes of A549 tumors were treated, and untreated tumors were 240.10 ± 40.01 and 186.70 ± 86.02 mm^3^ (*P* = 0.125), respectively. The mean changes in tumor volume were 7.92% ± 11.64% and 11.47% ± 6.16% (*P* = 0.739), respectively, on day 2; 25.91% ± 18.47% and 50.03% ± 36.22% on day 7 (*P* = 0.235), respectively; and 47.70% ± 28.19% and 91.93% ± 56.55% (*P* = 0.174), respectively, on day 14. A549 tumor volumes before and after treatment did not differ significantly between gefitinib-treated and untreated mice groups at these time points (*P* > 0.05).

### Changes in ^18^ F-FDG uptake on microPET

**Figure 2:**
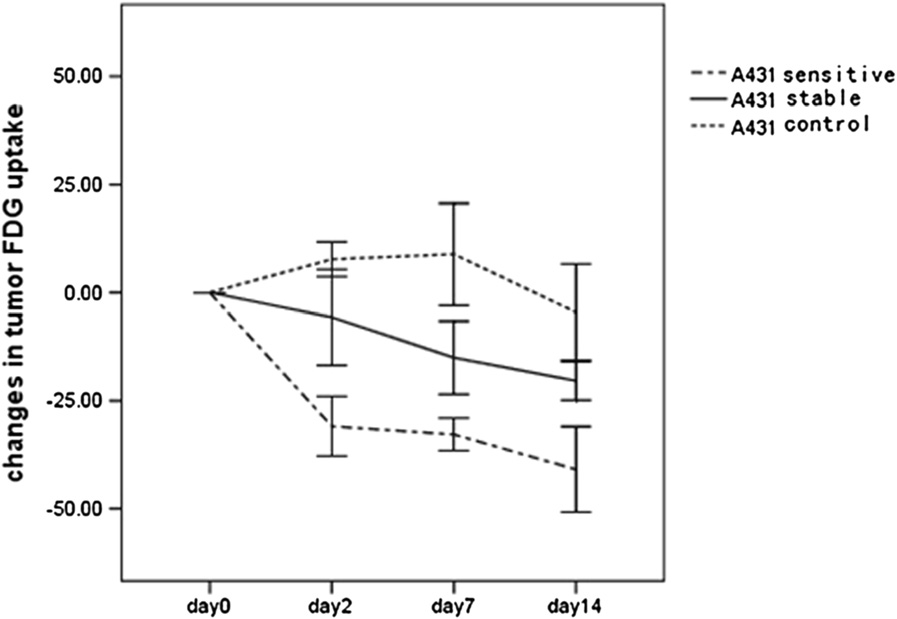
**Changes in A431 tumor** ^**18**^ **F-FDG uptake during treatment with gefitinib.** No significant differences were seen among the groups on day 0 (*P* > 0.05), although significant differences were observed on days 2, 7, and 14.

**Figure 3:**
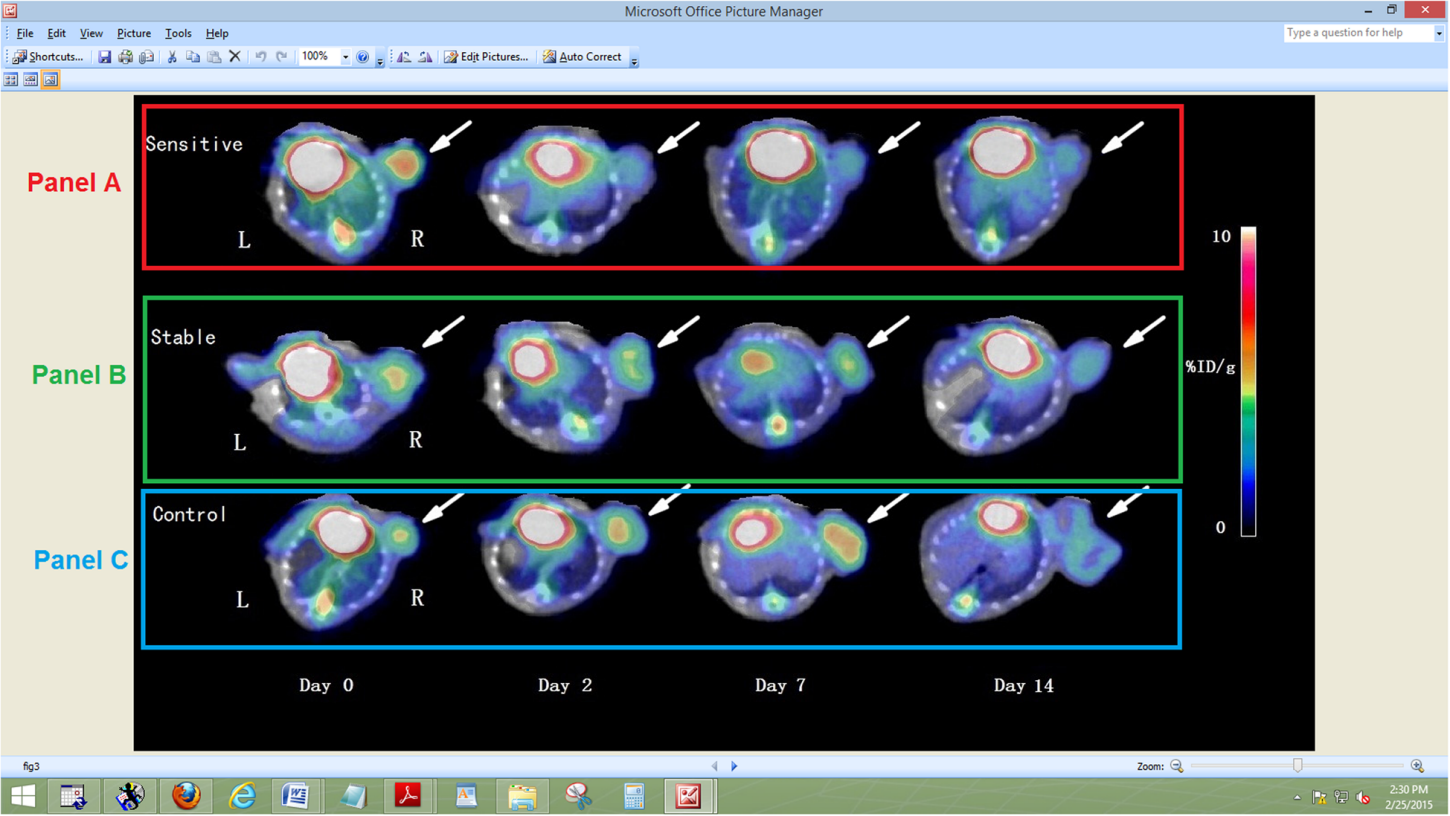
**MicroPET-CT imaging of A431 tumors before and after gefitinib treatment. (A)** Gefitinib-sensitive tumors on day 2, 7, and 14. **(B)** Gefitinib-stable tumors on day 2, 7, and 14. **(C)** Tumors in the control group on day 2, 7, and 14. White arrow indicates tumor.

^18^ F-FDG uptake by A431 and A549 tumors prior to treatment differed significantly (*P* = 0.001). Before treatment, ^18^ F-FDG uptake by A431 tumors sensitive and stable to gefitinib and by untreated tumors was similar (Figure 2). After 2 days of gefitinib treatment, the %ID/g_max_ decreased markedly in the gefitinib-sensitive group (−30.92% ± 6.66%) and slightly in the gefitinib-stable group (−5.68% ± 6.95%), but increased in the untreated group (7.72% ± 3.85%) (*P* = 0.000 for each pairwise comparison). At all time points, there were significant differences in changes of tumor ^18^ F-FDG uptake. %ID/g_max_ gradually increased in the untreated group, but decreased slightly after day 7 (Figure 3).

**Figure 4:**
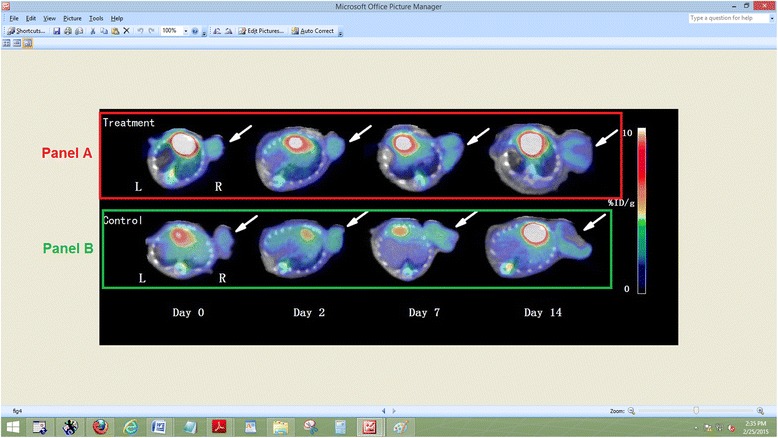
**MicroPET-CT imaging of A549 tumors before and after gefitinib treatment.** Tumors in the treated **(A)** and control **(B)** groups on day 2, 7, and 14. White arrow indicates tumor.

In contrast, ^18^ F-FDG uptake by A549 tumors did not differ significantly in gefitinib-treated and untreated mice (Figure 4). The %ID/g_max_ of A549 tumors in both groups gradually increased over time, but decreased slightly after day 7.

### ROC curve analysis of FDG uptake by A431 tumors

ROC curve analysis was performed to assess whether %ID/g_max_ or Δ%ID/g_max_ could discriminate between tumors sensitive and stable to gefitinib and to explore the possible threshold of %ID/g_max_ or Δ%ID/g_max_ that could accurately predict response to gefitinib. The Δ%ID/g_max_ of these tumors on days 2, 7, and 14 monitored treatment sensitivity (AUC = 1.000, *P* = 0.011 each). However, %ID/g_max_ of A431 tumors on days 2, 7, and 14 failed to differentiate between tumors sensitive and stable to gefitinib, with AUCs of 0.792 (*P* = 0.136), 0.667 (*P* = 0.394), and 0.813 (*P* = 0.110), respectively. Threshold with the maximum Youden index was used to calculate the sensitivity and specificity of this parameter. The cutoff point of Δ%ID/g_max_ on day 2 was −16.0%, which had a 100% sensitivity and a 100% specificity for predicting sensitivity to gefitinib.

### Semiquantitative analysis of histopathologic findings

On day 14, necrosis was obvious in all three groups of A431 tumors, but necrotic areas were significantly larger in gefitinib-sensitive (90.57% ± 4.77%) than in gefitinib-stable (79.90% ± 7.76%, *P* = 0.045) and untreated (69.04% ± 7.80%, *P* = 0.000) tumors, as well as being significantly larger in gefitinib-stable than in untreated tumors (*P* = 0.042). In A549 tumors, the mean percent necrosis on day 14 was similar in gefitinib-treated (66.95% ± 9.06%) and untreated (65.66% ± 9.24%) mice (*P* = 0.795).

## Discussion

Small-molecule EGFR-TKI drugs have shown survival benefits in certain patient subpopulations, including female gender, Asian ethnicity, non-smokers, adenocarcinoma, and specific mutations of the EGFR kinase domain [3,5]. Conversely, these characteristics are absent from a sizable subgroup of patients with tumors that do respond to EGFR-TKIs. Thus, it is very difficult to predict the outcomes of EGFR-TKI therapy based on patient and tumor factors alone. ^18^ F-FDG PET may therefore be an alternative method of predicting tumor response, by measuring early changes in tumor uptake of ^18^ F-FDG. This study showed that ^18^ F-FDG uptake by A431 xenografts decreased after only 2 days of treatment, consisting of two oral doses of gefitinib. After 14 days of treatment, the tumor volume of A431 xenografts decreased or remained stable, while the mean percent tumor necrosis differed significantly in gefitinib-treated and untreated mice. Conversely, treatment of gefitinib-resistant A549 xenografts resulted in an increase, not a decrease, in FDG uptake during the course of therapy, with the mean volume and percent necrosis of tumors not differing significantly in gefitinib-treated and untreated mice after 14 days. These findings suggest that decreased ^18^ F-FDG uptake, starting after only 2 days of treatment, could reflect the volumetric and pathological changes observed after 14 days.

SUV of ^18^ F-FDG has been used to differentiate between benign and malignant tumors and to monitor responses to various treatments [17]. Among the factors reported to correlate with SUV are tumor histological type, degree of differentiation, and tumor cell proliferation [18]. We also found that ^18^ F-FDG uptake by A431- and A549-generated tumor differed significantly before therapy. The volume of A431 tumor xenografts decreased or remained stable after 14 days of treatment, whereas the volume of A549 tumors was similar in gefitinib-treated and untreated mice. Both cell lines express wild-type EGFR, but this gene is amplified and overexpressed in A431 cells, explaining their higher drug sensitivity, whereas A549 cells have a Ras mutation that is responsible for resistance.

Changes in tumor ^18^ F-FDG uptake from baseline have been used to evaluate responses to treatment and predict survival. For example, chemotherapy-induced changes in glucose metabolism were found to be highly predictive of survival in 50 patients with advanced rectal carcinoma [19]. A study of 50 consecutive patients with locally advanced NSCLC who received induction chemotherapy or chemoradiotherapy found that a decrease in SUV from baseline to after three chemotherapy cycles was correlated with histopathologic response and prognosis [20]. Change in tumor ^18^ F-FDG uptake was a better predictor of prognosis than in tumor ^18^ F-FDG uptake itself, since the latter is a momentary measurement. Since ^18^ F-FDG uptake varies widely among patients, a comparison with pre-treatment ^18^ F-FDG uptake could be used to monitor changes in tumor metabolic activity. Similarly, we found that A431 tumor ^18^ F-FDG uptake on days 2, 7, and 14 did not differ significantly in tumors sensitive and stable to treatment and in untreated tumors, whereas changes in tumor ^18^ F-FDG uptake differed significantly in all three groups. These results showed that changes in ^18^ F-FDG uptake reflected the dynamic response of tumors to treatment and provided better and more personalized information than a single determination of ^18^ F-FDG uptake. Changes in ^18^ F-FDG uptake may be more reliable in assessing response to treatment, guiding individual treatments, and evaluating therapeutic efficacy and patient prognosis.

A study of changes in tumor ^18^ F-FDG uptake from before to after 7 days of preoperative neoadjuvant EGFR-TKI treatment in 23 patients with NSCLC found that a ≥ −25% change in ^18^ F-FDG uptake 7 days after treatment could predict tumor remission (histopathological response) and increased tumor necrosis [14]. Our histopathologic analysis showed that the necrotic areas of tumors sensitive and stable to gefitinib differed significantly from each other and from necrotic areas in untreated tumors. After 2 days of gefitinib administration, sensitive tumors showed a 30% decrease in metabolic activity, whereas stable tumors showed <10% decrease, indicating that changes in ^18^ F-FDG can predict pathological response and the effectiveness of gefitinib targeted therapy. Early changes in tumor ^18^ F-FDG uptake have been closely related with sensitivity to therapy and prognosis [21-23]. However, specific criteria to determine early ^18^ F-FDG PET response have not yet been defined, because of the involvement of many factors, such as tumor type, size, cell proliferation and diversity, time of evaluation, and inter-individual differences. A study of changes in ^18^ F-FDG uptake from before to after 14 days of neoadjuvant chemotherapy in patients with gastric cancer proposed a 35% reduction in ^18^ F-FDG uptake as a standard of treatment response and good prognosis [24]. The results of the PERCIST study recommended that a ≥30% decrease in tumor ^18^ F-FDG uptake after 4 weeks of treatment be a criterion for solid tumor remission [25]. Similarly, a ^18^ F-FDG PET evaluation of five NSCLC patients before and 2 days after the initiation of gefitinib therapy found that tumor uptake of ^18^ F-FDG was reduced 26% to 43% in patients who benefited from treatment [26]. An investigation of changes in ^18^ F-FDG uptake over 2 days in 20 patients with lung adenocarcinoma receiving gefitinib therapy found that a 25% cutoff of SUV reduction was not significantly associated with PFS (*P* = 0.095) [26], whereas a 20% cutoff showed a significant relationship between metabolic response and longer PFS (*P* < 0.0001). The threshold of ^18^ F-FDG uptake changes determined by ROC curve analysis in the current study was a 16.0% reduction on day 2. However, mean Δ%ID/g_max_ on day 2 was 7.72% ± 3.85% in untreated mice, similar to the coefficient of variation of ^18^ F-FDG uptake threshold and likely due to high experimental variability.

We observed that untreated tumors were several times larger on day 14 than before treatment, while tumor uptake had decreased after 7 days. Rapidly growing transplanted tumors may not be provided with sufficient blood supply by the host. However, metabolic uptake is associated with tumor blood supply, not tumor volume [27], which may explain the reduction in %ID/g_max_ on day 14.

Although these results indicate that ^18^ F-FDG PET can be used to monitor tumor response to EGFR-TKIs, several limitations should be noted. First, both the A431 and A549 cell lines express the wild-type EGFR. Additional studies are needed to assess mice bearing human tumors expressing EGFR with kinase domain mutations. Animal experiments provided a rigorous test of our hypotheses, but the clinical complexity of inter-individual differences and the cutoff value for ^18^ F-FDG uptake indicating clinical benefits from treatment required determination. However, threshold values are highly dependent on the number of subcutaneously injected tumor cells. For example, tumor cells expressing mutated EGFR may have different thresholds of changes in ^18^ F-FDG uptake at early time points. In addition, human cancers are more complex and heterogeneous than mouse tumor xenografts. Other clinically relevant factors may be associated with early changes in tumor ^18^ F-FDG uptake during gefitinib treatment. Therefore, changes in ^18^ F-FDG uptake may not be as dramatic in patients as in mouse tumor xenografts.

## Conclusions

Our findings suggest that response to gefitinib treatment, as assessed by ^18^ F-FDG PET, could help identify treatment sensitive tumors by their significant decrease in glucose metabolism. ^18^ F-FDG PET-CT is a sensitive and noninvasive method that may enable the selection of patients likely to benefit from gefitinib treatment. Confirming that reduced ^18^ F-FDG uptake could predict tumor response to gefitinib at early time points allowing the more sensitive detection of gefitinib-sensitive tumors and reduce drug side effects in clinical trials. Further studies, however, are needed to determine the optimal response criteria for various target therapies.
